# Toward mHealth Brief Contact Interventions in Suicide Prevention: Case Series From the Suicide Intervention Assisted by Messages (SIAM) Randomized Controlled Trial

**DOI:** 10.2196/mhealth.7780

**Published:** 2018-01-10

**Authors:** Sofian Berrouiguet, Mark Erik Larsen, Catherine Mesmeur, Michel Gravey, Romain Billot, Michel Walter, HUGOPSY Network, Christophe Lemey, Philippe Lenca

**Affiliations:** ^1^ IMT Atlantique Lab-STICC Université Bretagne Loire F-29238 Brest Brest France; ^2^ EA 7479 SPURBO Université de Bretagne occidentale Brest France; ^3^ Black Dog Institute University of New South Wales Sydney Australia; ^4^ Adult Psychiatry Brest Medical University Hospital at Bohars Brest France; ^5^ Sys.Vision Lannion France; ^6^ Réseau HUGOPSY Rennes France

**Keywords:** Suicide, Text Messaging, Electronic Health Records, Cell Phone, Secondary Prevention, Tertiary Prevention

## Abstract

**Background:**

Research indicates that maintaining contact either via letter or postcard with at-risk adults following discharge from care services after a suicide attempt (SA) can reduce reattempt risk. Pilot studies have demonstrated that interventions using mobile health (mHealth) technologies are feasible in a suicide prevention setting.

**Objective:**

The aim of this study was to report three cases of patients recruited in the Suicide Intervention Assisted by Messages (SIAM) study to describe how a mobile intervention may influence follow-up.

**Methods:**

SIAM is a 2-year, multicenter randomized controlled trial conducted by the Brest University Hospital, France. Participants in the intervention group receive SIAM text messages 48 hours after discharge, then at day 8 and day 15, and months 1, 2, 3, 4, 5, and 6. The study includes participants aged 18 years or older, who have attended a participating hospital for an SA, and have been discharged from the emergency department (ED) or a psychiatric unit (PU) for a stay of less than 7 days. Eligible participants are randomized between the SIAM intervention messages and a control group. In this study, we present three cases from the ongoing SIAM study that demonstrate the capability of a mobile-based brief contact intervention for triggering patient-initiated contact with a crisis support team at various time points throughout the mobile-based follow-up period.

**Results:**

Out of the 244 patients recruited in the SIAM randomized controlled trial, three cases were selected to illustrate the impact of mHealth on suicide risk management. Participants initiated contact with the emergency crisis support service after receiving text messages up to 6 months following discharge from the hospital. Contact was initiated immediately following receipt of a text message or up to 6 days following a message.

**Conclusions:**

This text message–based brief contact intervention has demonstrated the potential to reconnect suicidal individuals with crisis support services while they are experiencing suicidal ideation as well as in a period after receiving messages. As follow-up phone calls over an extended period of time may not be feasible, this intervention has the potential to offer simple technological support for individuals following discharge from the ED.

**Trial Registration:**

ClinicalTrials.gov NCT02106949; https://clinicaltrials.gov/ct2/show/NCT02106949 (Archived by WebCite at http://www.webcitation.org/6wMtAFL49)

## Introduction

### Brief Contact Intervention and Suicide Prevention

A previous suicide attempt (SA) is a strong predictor of death from future suicidal behaviors. Approximately one-third of individuals who attempt suicide seek treatment for their injuries from hospital emergency departments (EDs) [1], and the immediate period following discharge from hospital is critical for emergency and mental health care service follow-up, as most suicide reattempts occur within the first month of discharge [2]. For example, Hunt et al showed that 47% of fatal suicide reattempts occurred before the first scheduled follow-up appointment [3].

There has, therefore, been growing interest in the development of brief contact interventions (BCIs) delivered following discharge from the ED after an SA. BCIs are low-resource, nonintrusive interventions seeking to maintain long-term contact with patients after an SA. BCIs follow a structured schedule and remain operational over a sustained period of time. They commonly use short letters [4], postcards [5], phone calls [6], and crisis cards [7] to keep in contact with participants, without the provision of additional therapies. BCIs have been mostly used with clinical populations following presentation to an ED for self-harm, self-injury, self-poisoning or an SA. The content of BCIs differs between studies, but generally involves a short sentence expressing concern for the patient and emphasizing the availability of help should it be needed. BCIs have shown mixed or inconclusive results, but they indicate trends toward preventive effects in specific at-risk subgroups (eg, first suicide attempters, females, young suicide attempters) depending on the BCI employed. The pioneer intervention was proposed by Motto et al [4] and was based on postal contact. Motto provided 5 years of postal contact with patients who refused follow up after an SA. The objective was to show the patients that someone was concerned about their situation and maintain positive feelings toward them. After 5 years, a significant decrease in suicide-related deaths was observed in the contacted group (vs no-contact group). In a study by Carter et al [5], postcards were sent in the year following an SA, with a lower number of repeat episodes in the contacted group, especially among women. In general, these interventions aim to improve help-seeking and may facilitate access to health care services in the case of recurrence of suicidal ideation.

### Mobile Health and Suicide Prevention

By the end 2017, mobile cellular subscriptions worldwide are expected to reach 4.3 billion globally, and mobile broadband subscriptions have grown more than 20% annually in the last 5 years. Mobile phones are generally kept on at all times and carried everywhere, making them an ideal platform for the broad implementation of personalized and mobile health (mHealth) interventions [8]. mHealth has the potential to reduce waiting times for appointments and reduce the need to meet in-person with a clinician, successively diminishing the workload of mental health professionals; to be more cost-effective to practices; and to encourage self-care strategies [9]. A substantial amount of interactive and psychoeducational apps are readily available to download concerning a wide range of health issues, including suicide prevention [10]. A recent review of existing technology-enhanced interventions addressed determinants of suicidal behavior [9]. Included studies examined the use of standalone or, in most cases, adjunctive technology-enhanced interventions for suicide prevention delivered by mobile phone app, text message, telephone, computer, Web, CD-ROM, and video.

Mobile phones have many characteristics that make them well suited for health interventions. For example, the frequent use of mobile phones is associated with an opportunity for mass communication. mHealth can be defined as *the use of mobile computing and communication technologies in health care and public health* [11]. mHealth interventions have the potential to incorporate qualities often associated with more effective health communication interventions, such as personalization, tailoring, interactivity, and message repetition at a relatively low cost. In the suicide prevention setting, mobile phone technology can support the transition of care, specific treatment targets, and safety planning—all of which are important elements of treatment [12,13].

### Text Messages as a Suicide Prevention Brief Contact Intervention

Mobile text messaging (short message service, SMS) in particular has proven to be an effective form of psychiatric intervention [14]. Text messages can be sent in a standardized or individualized format and are available on all cellular phones, including low-cost devices. They can also be sent from a server-based platform that allows automatic prescheduling of message delivery and monitoring of delivery receipts. Studies such as Suicide Intervention Assisted by Messages (SIAM) [15] and Reconnecting AFTer a suicide attempt (RAFT) (Larsen et al, unpublished data, 2017) incorporate the use of stand-alone (RAFT) or adjunctive (SIAM) mobile phone-enhanced interventions for suicide prevention. The aim of these studies is to help connect participants with support services following discharge using SMS contacts, reducing repeat episodes of self-harm, reducing representations to the ED, and ultimately reducing deaths by suicide.

We conducted a feasibility and acceptability study of SIAM [16], demonstrating that the intervention was technically robust and well accepted by patients. A randomized controlled trial is ongoing; however, we hypothesize that a descriptive analysis of selected cases would bring insight regarding the capability of a mobile-based BCI to trigger patient-initiated contact with emergency services. This case series aimed to identify cases of patients recruited in the SIAM study that may demonstrate the capability of a mobile-based brief contact intervention for triggering patient-initiated contact with a crisis support team at various time points throughout the mobile-based follow-up period.

## Methods

### Study Design

We performed a descriptive analysis on a selected sample of patients randomized in the intervention group of the SIAM study, recruited between August 24, 2014, and June 5, 2017. SIAM is a 2-year, multicentre randomized controlled trial conducted by the Brest University Hospital, France. The study was registered on Clinical Trials Registry (clinicaltrials.gov NCT02106949). The study protocol is described in detail elsewhere (see Multimedia Appendix 1) [15]. The study includes participants aged 18 years or over, who have attended a participating hospital for an SA, and have been discharged from the ED or a psychiatric unit (PU) for a stay of less than 7 days. Eligible participants are randomized between the SIAM intervention messages and a control group. This study was approved by the French West VI ethics committee.

### Patients

Participants in the intervention group receive the SIAM text messages 48 hours after discharge, then at day 8 and day 15, and months 1, 2, 3, 4, 5, and 6. The messages refer to validation of the suffering, recall of the discharge agreement, and ongoing outreach care. The messages are also personalized with the participant’s name, the monitoring doctor’s name, and a local crisis telephone number. An example message contains the following text: “Mr X, we hope that your situation is getting better and that you can attend the consultation with Dr Y (April 7th 2011 at 10:00 h). You can call us for anything you may need at 0298000000.”

All participants, regardless of their randomization, received treatment as usual (TAU) including a postdischarge consultation with a psychiatrist. Study monitoring phone calls by a trained psychiatrist were also conducted at 6 and 13 months to perform follow-up evaluations.

The primary outcome measure of the SIAM study is the number of participants who have a repeat SA within 6 months. Secondary outcomes assess the number of attempts after 13 months and the number of deaths at both time points. To date, 387 participants have been recruited to the SIAM study. As recruitment is ongoing, complete data on the number of crisis calls initiated by participants in the intervention arm are not available. Nevertheless, representative cases were identified by the trial management office to reflect contacts initiated by participants at different points through the study.

Inclusion criteria for this case series were as follows: patients recruited in the SIAM study who had attended both the 6-month and 13-month monitoring interviews, and who had contact with emergency services during the study period.

Members of the study office (SB, CM, LC, and MW) established an initial list of patients meeting the inclusion criteria. The contents of the monitoring interviews were reviewed to identify patients who had contact with emergency services during the study period. SB and MC finally selected patients who demonstrated the capability of a mobile-based brief contact intervention for triggering patient-initiated contact with a crisis support team at various points throughout the follow-up period.

Three cases were identified, and we report the assessment of the participants’ baseline Mini International Neuropsychiatric Interview (MINI) [17] and Columbia Suicide Severity Rating Scale (CSSR-S) [18] and a narrative description of circumstances associated with their participant-initiated contact with the crisis team.

## Results

**Figure 1:**
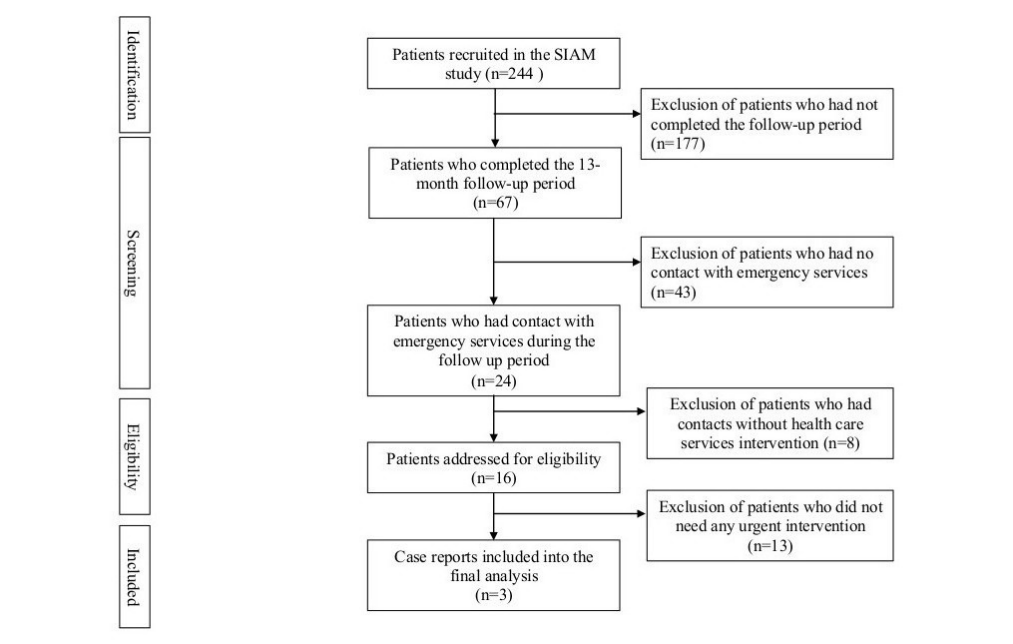
Flow of patient selection process.

Cases from the SIAM randomized controlled trial were identified to illustrate the impact of the mHealth brief contact intervention on further suicide ideation. The patient selection process for the case series is presented in Figure 1. Sociological main features of selected cases and history of SA are shown in Table 1. Psychiatric diagnoses are shown in Table 2.

*Case 1* was recruited to the study after an SA by deliberate poisoning. He had a history of previous SAs 2 and 6 years previously, also by deliberate self-poisoning (Table 1). He was diagnosed with general anxiety disorder and dysthymia (Table 2). He had no family history of mental disorder. He was divorced and unemployed. He was randomized in the intervention group. He also had acute alcohol intoxication (alcohol 2g/L) and a psychiatric history of hospitalization for alcohol withdrawal. This SA occurred 7 months after receiving a diagnosis of a chronic health condition (major life event) and 1 month after losing his job. He clearly expressed suicide ideation with a wish to die before the deliberate poisoning but not when we performed baseline assessment (Table 3). After discharge, an appointment with his general physician (GP) was scheduled.

The patient called the emergency service seeking help for suicide ideation. He told the nurse he had the emergency phone number from the last SMS he received (the second SMS received 3 days previously). After the phone call, he attended the appointment at the ED suggested by the nurse.

**Table 1:** Sociological main features and history of suicide attempt.

Patient	Sex	Age, years	Marital status	Employment	Total number of suicide attempts in lifetime	Number of suicide attempts in the last 3 years	Family history of mental disorder	Major life event
Case 1	Male	40-45	Divorced	Unemployed	2	1	No	Yes
Case 2	Male	55-60	In a relationship	Unemployed	4	2	Yes	No
Case 3	Female	40-45	In a relationship	Unemployed	5	3	No	No

**Table 2:** Psychiatric diagnosis of selected patients.

Patient	DSM^a^ diagnosis	Alcohol dependence and abuse
Case 1	Generalized anxiety disorder	Yes (both)
Case 2	Dysthymia	Yes (both)
Case 3	Dysthymia, generalized anxiety disorder	No (both)

^a^DSM: Diagnostic and Statistical Manual.

**Table 3:** Characteristics of the most recent suicide attempt.

Patient	Wish to be dead	How many times have you had suicidal thoughts?	When you have suicidal thoughts how long do they last?	Has there been a time when you started to do something to end your life but someone or something stopped you before you actually did anything?
Case 1	No	—^a^	—	—
Case 2	Yes	Less than once a week	Fleeting few seconds or minutes	Yes
Case 3	Yes	Less than once a week	Less than 1 hour	Yes

^a^— indicates missing data.

*Case 2* was recruited to the SIAM study after an SA by exsanguination. He was diagnosed with general anxiety disorder and dysthymia (Table 2). He was in a relationship and unemployed. He was randomized to the intervention group. The baseline evaluation indicated nonadherence to his medical treatment. He had a familial history of mental disorder (Table 1). The MINI assessment showed a diagnostic of dysthymia, alcohol abuse, and alcohol dependence (Table 2). The Columbia scale at baseline showed it was the fourth time he attempted suicide (Table 1). His most serious attempt occurred over 30 years ago when he was rescued from hanging. During the past few days, he had suicidal thoughts less than once a week. These thoughts usually lasted a few seconds (Table 3).

He received the ninth text message of the study and called the emergency service 6 days later. During the phone call, he disclosed suicidal ideation and that he had been drinking alcohol. He accepted the proposal to be driven to the ED by an emergency transport. He was subsequently admitted to a hospital for alcohol withdrawal.

*Case 3* was recruited after an SA by deliberate self-poisoning. She had a history of 5 previous SAs (Table 1). She was in a relationship and unemployed. The most recent attempt occurred in the context of a conflicting relationship with her adolescent son. Before the SA, she had suicidal thoughts less than once a week. These thoughts usually lasted less than an hour (Table 3).

The MINI scale at baseline showed a personal history of dysthymia. The Columbia scale at baseline showed her first SA occurred 4 years ago. After discharge, an appointment with her GP was scheduled. The patient received the fifth text message on a day when she was experiencing intense suicidal ideation. Immediately after receiving the message, she called the phone number provided in the message. She got in touch with the emergency service that proposed an immediate intervention of an emergency transport. Within 1 hour of receiving the message, she was admitted to the emergency service.

## Discussion

### Principal Findings

We have presented three cases describing situations within the SIAM intervention where crisis support services offered through this BCI have been initiated. In each case, the contact has been initiated by the study participant immediately after receiving a message (Case 3) or a few days later (Case 1 and 2). These contacts have also been initiated over a range of periods since discharge, from 1 week (Case 1) to 6 months (Case 2). These cases highlight the potential for connecting individuals to crisis services after an SA using automated text messages. The use of text messaging is likely to be more cost-effective than attempting follow-up phone calls, which require considerable on-going resources that may limit the feasibility of regular follow-ups. Furthermore, our experience from other studies indicates that calls are frequently not answered [19].

The sociodemographic details in Table 1 indicate that all the 3 cases relate to individuals aged over 40 years, and all the 3 were unemployed. This is perhaps surprising, as mHealth interventions are often considered to have the greatest potential for younger participants. These results demonstrate the potential for such interventions across a range of sociodemographic characteristics.

This case series presents an example of a real-world intervention triggered by an mHealth intervention. In particular, this intervention aims to strengthen the connection between patients and their care team by encouraging contact during a crisis. Similar low-intensity interventions also have the potential to be made available to a wider population of participants, for example, through other health care services or self-registrations from individuals who may be in crisis but not otherwise seeking help. There is, therefore, a potential to connect more people with appropriate care before a hospital presentation.

### Limitations

Although these cases demonstrate the feasibility of initiating crisis contact using text messages, the effectiveness cannot yet be determined as the SIAM study is ongoing. It is therefore too early to address whether a text message–based BCI can reduce repeat episodes and mortality. Furthermore, the comparator arm in this study is TAU; therefore, the effectiveness of a text message–based BCI versus a telephone-based BCI cannot be compared. Further investigation into the methods [20] and mechanisms [21] of BCIs is warranted.

The SIAM mobile intervention was proposed as an adjunct to existing treatment strategies and not as a substitute. It was not designed as a remote counseling service [22].

As a result, the text message–based contact was deliberately limited to a one-way communication. We disabled the feature of two-way communication to encourage phone calls and face-to-face contacts. However, as illustrated, a simple message can have an important impact. Contents of messages are of particular importance, and certain key characteristics such as personalization, caring sentiment, and polite text are associated with more successful preventative messages [23].

As with any outreach activity, this intervention presents a possible risk of intrusiveness into the daily lives of participants. This issue has not been assessed in the papers we reviewed or in other reviews in the field [24], and in our earlier feasibility study, no patients reported finding this mHealth intervention intrusive [16]. Conversely, in the third case reported here, a text message was received at a timely moment. However, an SMS may also arrive at an inconvenient time; therefore, informational messages may be ignored or deleted. Extended interventions incorporating momentary assessments may additionally lack responses at these times. Researchers should ensure that such burden would not be detrimental to participants' well-being, particularly when studying individuals who have recently been discharged or may currently be facing a crisis.

In our study, the SMS intervention was proposed as an adjunct of TAU. Others have suggested additional supportive outreach and coping strategies; for example, in the Brief Mobile Treatment (BMT) intervention [25], a patient received generic weekly text messages up to 26 weeks, supportive phone calls, and access to audio phone messages to reinforce psychotherapy principles. BMT participants received text reminders about meditation, problem solving, spiritual or philosophical ideas, the importance of social support, avoiding alcohol and drugs, and crisis helpline details. Our eventual aim is to integrate an mHealth intervention within existing emergency care procedures, as this may increase the effectiveness of both the mHealth intervention and emergency services.

The Web app we developed for the SIAM study allowed the patient to respond to messages they received. However, as part of our safety protocol to avoid the possibility of unanswered crisis messages, we disabled the two-way communication feature. Since we started the study, natural language processing (NLP) machine learning prediction methods have been shown to predict suicide risk as well as heightened psychiatric symptoms in free-text responses sent via a mobile phone [26]. NLP methods applied on SMS communication may help to create low-cost and effective alternatives to traditional resource-heavy data monitoring systems and support decision making of clinicians.

SMS is a powerful tool to connect the patient with health care services. Being a simple and affordable technology, it also allows for the addition of other content as links to support websites and self-monitoring apps. A recent review showed that mHealth interventions had a larger effect when used for conveying psychoeducation. Safety planning, for example, can be easily implemented [27]. Furthermore, assessment of symptoms could also be performed using SMS, and it may allow for an accurate assessment of suicide ideations [28]. These features may help to identify, together with face-to-face assessment, personalized trajectories of symptoms, cognitive abilities, and symptoms impacting other symptoms [29].

The use of SMS allows the health care team to keep in touch with people that are not reachable by existing BCIs. It may be used as a useful additional or alternative to standard care when people decline other forms of treatment. Although we used a Web system to deploy our intervention, our main goal is to encourage person-to-person contact between suicide attempters and health care providers. Torous et al [30] evaluated an mHealth intervention that successfully encouraged participants to be more open when disclosing symptoms of depression. We believe that our mHealth intervention may also improve the likelihood of patients disclosing suicide ideation to emergency services and seeking help. The full effects of the SIAM intervention and related results on suicide reattempt reduction will be reported upon conclusion of the trial.

### Conclusions

Brief contact interventions are a promising technique for maintaining contact with patients following discharge from the ED and preventing repeat SAs. This case series has demonstrated the ability of text message BCIs to encourage patients to contact health care services in times of a crisis and over periods up to 6 months following discharge from the hospital. An ongoing randomized controlled trial of the SIAM intervention aims to demonstrate the effectiveness of such mHealth BCIs for suicide prevention.

## Appendix

Multimedia Appendix 1 Siam protocol description video.

Multimedia Appendix 2 CONSORT checklist.

## Abbreviations

BCIs: brief contact interventions
BMT: Brief Mobile Treatment
CSSR-S: Columbia Suicide Severity Rating Scale
ED: emergency department
GP: general physician
mHealth: mobile health
MINI: Mini International Neuropsychiatric Interview
NLP: natural language processing
PU: psychiatric unit
RAFT: Reconnecting AFTer a suicide attempt
SA: suicide attempt
SIAM: Suicide Intervention Assisted by Messages
SMS: short message service
TAU: treatment as usual
